# Supplementary material for: Generation of a Triple-Shuttling Vector and the Application in Plant Plus-Strand RNA Virus Infectious cDNA Clone Construction
Source: Int J Mol Sci. 2023 Mar 13;24(6):5477. doi: 10.3390/ijms24065477 (PMC10056883; doi:10.3390/ijms24065477)
Supplement: Supplementary file 1 [file ijms-24-05477-s001.zip › Supplementary Materials.pdf]

Supplementary Table S1. Primer used in the vector construction and RT-PCR detection.

Primer Name	Sequence (5'-3')	Length of the amplicon (bp)	Tm (℃)	Purpose (Amplification)
pGBK-2686F1	gttgctgcctgtgatcaCCGCGGCACATTTCCCCGAAAAGTGCCACC	2,686	62	Yeast 2μ replication origin element
pGBK-2686R1	cgacggagccgattttgaaaCCGCGGGCGGTATTTTCTCCTTACGCATCTGTGC		61	
CMV-Fny/RNA1&RNA2/yeast/StuI/F	gttcatttcatttggagaggGTTTATTTACAAGAGCGTACGGTTCAATCC	3,357	60	The cDNA of CMV RNA1
CMV-Fny/RNA1/yeast/BamHI/R	catccggtgacagggtatcgTGGTCTCCTTTTAGAGACCCCCACGAAAG		64	
CMV-Fny/RNA2/yeast/BamHI/R	catccggtgacagggtatcgTGGTCTCCTTTTGGAGGCCCCACAAAAG	3,050	65	The cDNA of CMV RNA2, combine with the third primer
CMV-Fny/RNA3/yeast/StuI/F	gttcatttcatttggagaggGTAATCTTACCACTGTGTGTGTGCGTG	2,216	61	The cDNA of CMV RNA3
CMV-Fny/RNA3/yeast/BamHI/R	catccggtgacagggtatcgTGGTCTCCTTTTGGAGGCCCCCACG		66	
CMV-Fny/CP/F	ATGGACAAATCTGAATCAACC	657	53	The CMV CP encoding sequence
CMV-Fny/CP/R	TCAAACTGGGAGCACCCCAGA		61	
YoMV(1-2991)/yeast/StuI/F	gttcatttcatttggagaggGTTGTTGCAATAAAAATAAAAC	2,991	52	The fragment 1 of YoMV
YoMV(1-2991)/R	TACACATCACTGATCCATCGT		55	
YoMV(2971-6305)/F	ACGATGGATCAGTGATGTGTA	3,335	55	The fragment 2 of YoMV
YoMV(2971-6305)/yeast/BamHI/R	catccggtgacagggtatcgTTGGGCCCCTACCCGGGGTTA		64	
YoMV/CP/F	ATGGTTTACAACATCACGAGCTC	474	57	The YoMV CP encoding sequence
YoMV/CP/R	TGTAGCTGGCGCAGTAGCCCAA		63	
ReMV(1-2159)/yeast/StuI/F	gttcatttcatttggagaggGTATTTTTTAACAACAATTACC	2,159	49	The fragment 1 of ReMV
ReMV(1-2159)/R	CACAATCGAGCTCATCTGTTTGCG		60	
ReMV(2136-4296)/F	CGCAAACAGATGAGCTCGATTGTG	2,161	60	The fragment 2 of ReMV
ReMV(2136-4296)/R	GTCTCCGCCAGATTTCGTACTC		58	
ReMV(4275-6395)/F	GAGTACGAAATCTGGCGGAGAC	2,121	58	The fragment 3 of ReMV
ReMV(4275-6395)/yeast/BamHI/R	catccggtgacagggtatcgTGGGCCCCTACCGGGGGTAACGGGGGAATTC		69	
ReMV/CP/F	ATGTCTTATACAATTGCAACTCC	480	52	The ReMV CP encoding sequence
ReMV/CP/R	TCAAGTTGCGGGACCAGAAGTC		60	

Note: the small letters indicate the adaptor sequences for the yeast HR-based assembly.

A.

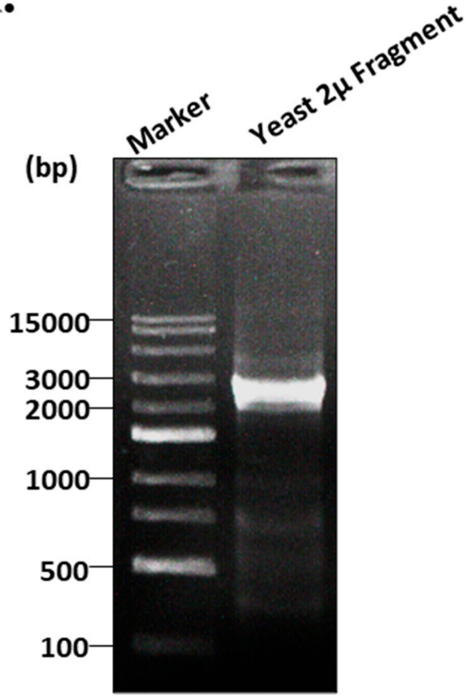

Supplementary Figure S1. Agarose gel electrophoresis of the yeast 2µ replication origin and the tryptophan (TRP1) autotrophic marker gene fragment.
